# Cold Plasma Treatment as an Alternative for Ochratoxin A Detoxification and Inhibition of Mycotoxigenic Fungi in Roasted Coffee

**DOI:** 10.3390/toxins11060337

**Published:** 2019-06-13

**Authors:** Paloma Patricia Casas-Junco, Josué Raymundo Solís-Pacheco, Juan Arturo Ragazzo-Sánchez, Blanca Rosa Aguilar-Uscanga, Pedro Ulises Bautista-Rosales, Montserrat Calderón-Santoyo

**Affiliations:** 1 Laboratorio Integral de Investigación en Alimentos, Tecnológico Nacional de México/Instituto Tecnológico de Tepic, Av. Tecnológico #2595, Col. Lagos del Country, C.P. 63175 Tepic, Nayarit, Mexico; mcapalomaa@outlook.es (P.P.C.-J.); arturoragazzo@hotmail.com (J.A.R.-S.); 2 Laboratorio de Microbiología Industrial, Centro Universitario de Ciencias Exactas e Ingeniería, Universidad de Guadalajara, Boulevard Marcelino García Barragán #1421, Col. Olímpica, C.P. 44430 Guadalajara, Jalisco, Mexico; josuesolisp@gmail.com (J.R.S.-P.); agublanca@gmail.com (B.R.A.-U.); 3 Centro de Tecnología de Alimentos, Universidad Autónoma de Nayarit, Ciudad de la Cultura “Amado Nervo”, C.P. 63155 Tepic, Nayarit, Mexico; u_bautista@hotmail.com

**Keywords:** roasted coffee, mycotoxigenic fungi, ochratoxin A, cold plasma, detoxification, brine shrimp bioassay

## Abstract

Ochratoxin A (OTA) produced by mycotoxigenic fungi (*Aspergillus* and *Penicillium* spp.) is an extremely toxic and carcinogenic metabolite. The use of cold plasma to inhibit toxin-producing microorganisms in coffee could be an important alternative to avoid proliferation of mycotoxigenic fungi. Roasted coffee samples were artificially inoculated with *A. westerdijikiae, A. steynii, A. versicolor*, and *A. niger*, and incubated at 27 °C over 21 days for OTA production. Samples were cold plasma treated at 30 W input power and 850 V output voltage with helium at 1.5 L/min flow. OTA production in coffee was analyzed by high performance liquid chromatography coupled to a mass spectrometer (HPLC-MS). After 6 min of treatment with cold plasma, fungi were completely inhibited (4 log reduction). Cold plasma reduces 50% of OTA content after 30 min of treatment. Toxicity was estimated for extracts of artificially contaminated roasted coffee samples using the brine shrimp (*Artemia salina*) lethality assay. Toxicity for untreated roasted coffee was shown to be “toxic”, while toxicity for cold plasma treated coffee was reduced to “slightly toxic”. These results suggested that cold plasma may be considered as an alternative method for the degradation and reduction of toxin production by mycotoxigenic fungi in the processing of foods and feedstuffs.

## 1. Introduction

Ochratoxins belong to a family of structurally related, secondary fungal metabolites produced by various *Penicillium* and *Aspergillus* strains. Among them, ochratoxin A (OTA) is reputed to be important mycotoxin in coffee. OTA possesses properties such as nephrotoxicity, carcinogenicity, and teratogenicity and is classified as carcinogenic for humans (group 2B) [1,2]. According to the Commission of European Communities 2006, the maximum accepted levels for OTA are 5 µg/kg in roasted coffee beans and ground roasted coffee, and 10 µg/kg in soluble coffee. Some studies have revealed that the roasting treatment of green coffee at 200 °C for 20 min reduced the OTA levels by only 0–12% [3]. OTA binds to the plasma proteins [4], and then OTA could be bound to the coffee proteins. Changes to OTA during heat treatment have been studied; a partial isomerization of OTA in position C3 into a diastereomer occurred at high temperatures [5]. Therefore, the relative OTA thermo-stability could cause toxicological problems during the consumption of coffee products.

Several strategies are available for the detoxification of mycotoxins and inhibition of fungi growth, which include methods such as thermal inactivation, irradiation, ultrasound treatment, and biological control agents [6]. Although these are widely used methods, they are time consuming and result in reductions in the nutritional quality of food products, variations in sensorial characteristics, and higher costs can occur.

Cold plasma is an innovative technology with potential for the inactivation of various pathogenic and spoilage microorganisms and the detoxification of foods [7]. Cold plasma is the fourth state of matter, being an ionized gas containing a rich mixture of reactive neutral species, energetic charged particles, UV photons, and intense transient electric fields, which can interact simultaneously and synergistically with the food [8]. Ionization is the first important step for plasma chemistry. Plasma chemistry depends on several factors such as feed gas composition, relative humidity, the power supplied, and treatment time [9]. Various studies have shown the potential of cold plasma to inactivate toxigenic fungi, leading to the detoxification of hazelnuts, peanuts, pistachios, and nuts [6,10], date palm fruits [11], and groundnuts [9]. Solís-Pacheco et al. [12] carried out a study on the sterilization of cinnamon and camomile to eliminate fungi and yeasts by the use of cold plasma. These authors obtained reductions higher than 1.0 log CFU/g at 750 and 850 V for chamomile and 0.68 ± 0.18 log CFU/g for cinnamon at 850 V after 10 min of treatment. They concluded that the best treatment to obtain significant reductions of yeast and mold counts in chamomile and cinnamon samples with the lowest degradation of antioxidant compounds was the application of plasma energy at 750 V for 10 min.

Therefore, the objective of this study was to explore cold plasma as a technology to inhibit toxigenic fungi and to detoxify OTA in roasted coffee, as well as to demonstrate the reduction in toxicity for the cold plasma treated samples.

## 2. Results

### 2.1. Inhibition of Fungal Spores in Roasted Coffee by Cold Plasma Treatment

**Figure 1:**
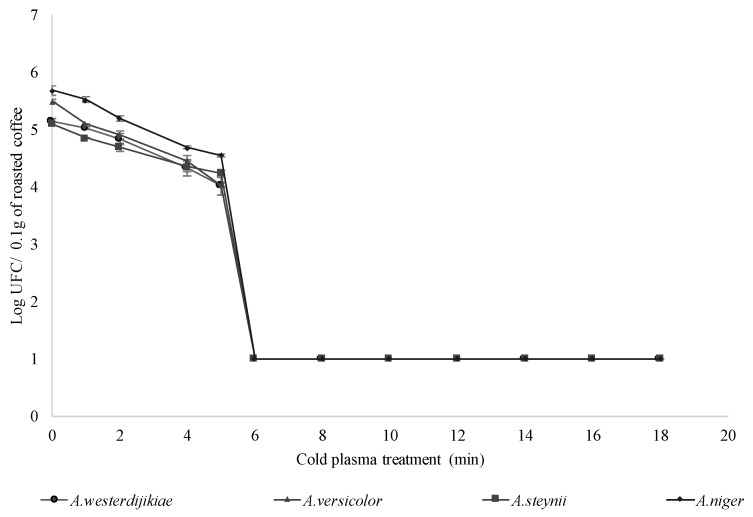
Logarithmic survival curves of *A. westerdijikiae, A. steynii, A. versicolor*, and *A. niger* spores on roasted coffee samples cold plasma treated (30 W input power, 850 V output voltage, He-air mixture at 1.5 L/min).

A complete inhibition of *A. westerdijikiae, A. steynii, A. versicolor*, and *A. niger* spores was achieved (4 log reduction) after 6 min exposure to cold plasma treatment (*p* < 0.05) (Figure 1; Table S1). Méndez-Vilas [13] reported damage in the microbial cellular membrane due to its interaction with active species and a subsequent loss of cytoplasmic material as the final cause of microorganism death. Additionally, DNA molecules—highly sensitive to ions, neutral species, and UV light—can be altered when these active species penetrate and reach the nucleus [14,15].

### 2.2. Effect of Cold Plasma on Detoxification of OTA Produced in Roasted Coffee by Mycotoxigenic Fungi

OTA production in roasted coffee artificially contaminated and incubated over 21 days was determined as follows: *A. westerdijikiae* 96.45 µg/kg, *A. steynii* 67.79 µg/kg, *A. niger* 91.03 µg/kg, and *A. versicolor* 76.74 µg/kg. After 30 min of cold plasma exposure, OTA was reduced by approximatively 50% (*p* < 0.05) (Table 1; Table S1). These results suggest the application of cold plasma may have a strong potential not only in mycotoxin degradation, but also in fungi control, and subsequently in the reduction of toxin production by mycotoxigenic fungi, thus suggesting this process can be effectively used in industrial food production. Despite an absence of OTA total reduction, the achieved reduction of 50% could be high enough to meet the requirements stablished by the European Union legislation which established the maximum accepted levels for OTA at 5 µg/kg for roasted coffee beans and ground roasted coffee and 10 µg/kg for soluble coffee [16]. Therefore, this technique can be promising for mycotoxin detoxification in the coffee industry as an alternative to preventing or eliminating OTA and toxigenic fungi. 

The effect of cold plasma on OTA detoxification could be attributed to the reactive gas species generation of such ions as (H_3_O^+^, O^+^, O^−^, OH^−^, N_2_^+^), molecular species (O_3_, H_2_O_2_), and reactive radicals (O•, OH•, NO•), as well as to UV irradiation and etching [7,17]. These species could promote the opening of the chlorophenolic group containing a dihydro-isocoumarin and/or amide-linked to L-phenylalanine.

Some studies have reported the efficacy of cold plasma on detoxification of mycotoxins in food products. Basaran et al. [6] indicated a 50% reduction in aflatoxins (AFB1, AFB2, AFG1, and AFG2) after 20 min exposure to air plasma, while only a 20% reduction was obtained after 20 min of exposure to sulfur hexafluoride plasma. Ouf et al. [11] demonstrated synthesis inhibition of fumonisin B2 and ochratoxin A by *A. niger* after treatment for 6 and 7.5 min with argon plasma jet. Devi et al. [9] showed 70% and 90% reductions on groundnuts samples treated with air plasma at 60 W (12 min) and 40 W (15 min), respectively.

Atalla et al. [16] explained the aflatoxin B1 reduction by UV and fluorescent light in terms of degradation of aflatoxin crystals mediated by their high sensitivity to UV radiation and changes in the structure of the terminal furan ring. During cold plasma generation, UV radiation is an important component for the detoxification of mycotoxins.

### 2.3. Sensitivity of Brine Shrimp Larvae to OTA

According to the obtained results for OTA detoxification by cold plasma, it was desirable to study the toxicity of structures resulting from the application of this technology. Brine shrimp incubated with untreated OTA extracts showed a toxicity level classified as “toxic”. On the other hand, larval mortality for cold plasma treated OTA extracts was significantly reduced, and a toxicity level of “slightly toxic” was obtained for all samples (Table 2). Mycotoxin detoxification is mediated by the union of free radicals to the heterocyclic rings in their molecule [17]. In this way, when mycotoxins are irradiated, three possible results can be obtained: first, the resulting structures are more toxigenic than the original molecule; second, the resulting molecules are equally toxigenic as the original toxin; and third, the resulting fragments present lower toxicity compared with the original toxin molecule [18]. Ultimately, in this study, a lower degree of toxicity was obtained when OTA was cold plasma treated.

## 3. Conclusions

Cold plasma is a novel non-thermal technology that has shown significant potential for applications in food industries for food safety and shelf life extension. The results presented here suggest this method could be a viable option for commercial application in the food industry. The effect of cold plasma treatment on ochratoxin A contained in roasted coffee can be considered promising in terms of safety and process efficiency. However, the application of cold plasma needs further extensive research to reveal the mechanisms of detoxification.

## 4. Materials and Methods 

### 4.1. Biological Materials

Roasted ground coffee was procured from a local market in Nayarit, Mexico. Coffee samples were autoclaved for 20 min at 121 °C and 15 psi, and dried in a laminar flow hood (CFLV-120, NOVATECH) for 1 h.

OTA producing strains of *A. westerdijikiae* (KP329736.1), *A. steynii* (FM956458.1), *A. niger* (JN226991.1), and *A. versicolor* (NR131277.1) were isolated from roasted coffee (*Coffea arabica* L.) from a coffee-growing region of Nayarit, Mexico [19]. Individual fungal cultures were grown on potato dextrose agar (PDA) for 7 days at 28 °C. Spore suspension was prepared by flooding the mycelia surface with 10 mL of sterile distilled water. The suspension was filtered and the spores counted using a Neubauer camera. The suspension was adjusted to a final value of 5 × 10^5^ spores mL^−1^.

### 4.2. Plasma Generator

The plasma apparatus was designed and built at the Laboratory of Plasma Physics of the National Nuclear Research Institute in Toluca, Mexico. The plasma reactor was described by Solis-Pacheco et al. [12].

### 4.3. Inhibition of Toxigenic Fungi Using Cold Plasma Treatment on Roasted Coffee

Samples of 0.5 g of sterilized roasted coffee were inoculated with 5 µL of a spore’s suspension (5 × 10^5^ spores g^−1^) and rested for 30 min to facilitate proper attachment of spores on coffee particles. Samples were allowed to dry in a laminar flow hood. Afterwards, the samples were exposed to plasma using commercial helium gas (Praxair, Mexico) at a constant flow of 1.5 L/min, applied at 850 volts for different periods of time (0, 1, 2, 4, 5, 6, 8, 10, 12, 14, 16, and 18 min). Before and after plasma treatment, the enumeration of fungi (log CFU/g) were determined. The samples were diluted in 1 mL of 0.1% peptone water and homogenized for 10 s. Decimal dilutions were performed and pour plated with potato dextrose agar (PDA, Bioxon, Mexico) containing 2% ampicillin (Bayer, México) and 0.6% Bengal rose (Analytyka, Mexico). All plates were incubated at 28 °C for 48 h. Fungi colonies were enumerated and results reported in log CFU/g of sample. Each experiment was conducted in triplicate, and the whole analysis was performed in duplicate.

### 4.4. OTA Production by Mycotoxigenic Fungi and Detoxification by Cold Plasma

Isolated fungi were inoculated in the center of a plate containing PDA medium and incubated at 27 °C for 5 days. One gram of roasted coffee was added to 3 mL of distilled water and autoclaved for 15 min at 121 °C and 15 psi. After cooling, samples were inoculated with 3 agar disks taken from the periphery of the colony [20]. Once inoculated, the roasted coffee samples were incubated at 27 °C for 21 days. The exposure times of cold plasma for detoxification of OTA in the samples of coffee were 0, 1, 4, 8, 12, 16, 20, 24, and 30 min, using commercial helium gas at 1.5 L/min, 30 W input power, and a voltage of 850 V.

### 4.5. OTA Detection by HPLC-MS

**Figure 2:**
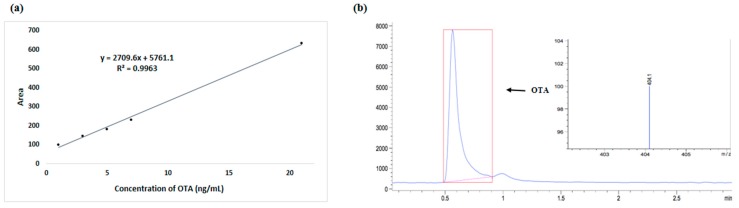
OTA standard curve (1–21 ng/mL) (**a**), OTA chromatogram (0.021 ng/mL) by HPLC-MS (**b**).

At the end of the plasma treatment, extraction was carried out in an alkaline solvent (pH 9.4) consisting of 3% sodium bicarbonate in methanol/water (20/80, *v*/*v*), and the suspension was homogenized for 50 min at 60 °C. The extract was centrifuged (model EBA 21, Heittich) at 1036.39× *g* for 5 min. Ten milliliters of filtered extract were diluted in 40 mL phosphate buffer (pH 7.0, 0.1 M). The mixture was purified using an immunoaffinity column (Ochrastar R) purchased from Romer Labs (Tulin, Austria). OTA was eluted by 6 mL of methanol and evaporated to dryness in a rotary evaporator [21]. The residue was re-suspended in 1 mL of the mobile phase (water/acetonitrile, 50:50). Quantification was done by HPLC-MS (Agilent Technologies). The operating conditions were as follows: mobile phase of 5 mM ammonium acetate/acetonitrile (65/35, *v*/*v*) at 40 °C with a flow rate of 0.2 mL/min for 4 min run. A column Agilent Technologies Zorbax SB-C18 (2.1 × 50 mm id: 1.8 μm) was used. The detection of OTA was performed in a HPLC-MS system model 6120 series LC quadrupole equipped with an electrospray ion (ESI) MS (Agilent Technologies) in ionization mode and positive mode (capillary 3500 V, nebulizer 25 psi (nitrogen), under dry gas nitrogen at 9 L/min, at 350 °C, and the fragmentor voltage set at 95 V; ion monitoring SIM selected, *m*/*z* 404 (OTA). A standard curve prepared with different OTA concentrations (1, 3, 5, 7, and 21 µg/mL) in acetonitrile was used to determine OTA in coffee samples (Figure 2).

### 4.6. Brine Shrimp Bioassay

Brine shrimp eggs (*Artemia salina*) were incubated in artificial seawater illuminated by artificial light (60 W lamp) and gently aerated for 24 h at 28 °C. Afterwards, the nauplii (larvae) were collected by pipette from the lighted side, whereas their shells were left in the dark side. The test samples (extract from roasted coffee inoculated with toxigenic fungi) were prepared in acetonitrile-water (50:50). The actual concentrations of the whole solutions were determined by HPLC-MS analyses (quantitation based on OTA as internal standard) and considered with the data evaluation. Filter paper disks (d = 0.5 cm) were submerged in microtubes containing 30 µL of OTA extracts and air-dried. Then, each disc was transferred into a 6-well microtitrate plate containing artificial sea water (2 mL) and 10 nauplii; plates were maintained at room temperature for 48 h under light and dead larvae were counted by examination under a binocular stereoscope. Discs submerged in acetonitrile-water solvent (50:50) were used as controls. Mortality was determined according to Equation (1).

(1) $$\%\;\mathrm{deaths}=\;\frac{\mathrm{Dead}\;\mathrm{nauplii}\;\mathrm{in}\;\mathrm{the}\;\mathrm{biossay}\;}{\mathrm{Nauplii}\;\mathrm{used}\;\mathrm{in}\;\mathrm{the}\;\mathrm{biossay}-\mathrm{Dead}\;\mathrm{nauplii}\;\mathrm{in}\;\mathrm{the}\;\mathrm{control}}\times 100$$

OTA toxicity was rated as follows: 0–9% mortality, nontoxic (NT); 10–49% mortality, slightly toxic (ST); 50–89% mortality, toxic (T); 90–100% mortality, very toxic (VT) [22].

### 4.7. Statistical Analysis

Statistical analysis was performed using STATISTICA version 9.0. Analysis of variance (ANOVA) and multiple comparison procedures (least significant difference–LSD) test were conducted to determine whether there were significant differences (*p* < 0.05) among treatments for spores’ inhibition or OTA detoxification.

## Figures and Tables

**Table 1:** Effect of cold plasma applied for different periods of time on ochratoxin A (OTA) detoxification of roasted coffee.

Exposure Time (min)	OTA (µg/kg)							
	*A. westerdijikiae*	Reduction (%)	*A. steynii*	Reduction (%)	*A. niger*	Reduction (%)	*A. versicolor*	Reduction (%)
0	96.45 ± 3.08 ^a^		67.69 ± 3.66 ^a^		91.03 ± 12.06 ^a^		76.741 ± 10.12 ^a^	
1	89.86 ± 4.43 ^a^	6.83	64.57 ± 1.92 ^ab^	4.61	87.31 ± 3.56 ^a^	4.09	76.61 ± 1.91 ^a^	0.17
4	81.34 ± 16.91 ^ab^	15.67	59.07 ± 4.97 ^bc^	12.73	72.81 ± 1.99 ^b^	20.02	75.44 ± 10.96 ^a^	1.69
8	77.17 ± 14.79 ^bac^	19.99	55.17 ± 1.63 ^dc^	18.50	71.77 ± 1.28 ^b^	21.16	69.19 ± 22.76 ^a^	9.84
12	73.62 ± 8.43 ^bac^	23.67	49.48 ± 5.49 ^de^	26.90	69.50 ± 2.86 ^b^	23.65	68.36 ± 2.38 ^a^	10.91
16	62.89 ± 22.09 ^bdc^	34.80	43.48 ± 1.75 ^ef^	35.37	66.4 ± 1.99 ^cb^	27.06	67.08 ± 8.85 ^a^	12.59
20	51 ± 5.26 ^bdc^	47.12	41.42 ± 0.69 ^f^	38.31	65.37 ± 9.97 ^cb^	28.19	63.67 ± 1.29 ^a^	17.02
24	54.39 ± 1.08 ^dc^	43.61	38.28 ± 1.59 ^fg^	43.45	63.76 ± 3.40 ^cb^	29.96	63.28 ± 13.36 ^a^	17.53
30	38 ± 4.20 ^d^	60.60	31.39 ± 0.03 ^g^	52.86	54.34 ± 0.85 ^c^	40.31	51.18 ± 6.64 ^a^	33.31

Values followed by the same letter in the columns are not significantly different.

**Table 2:** Toxic activity of untreated and 30 min cold plasma treated coffee extracts against brine shrimp.

Mycotoxigenic Fungi in Coffee	OTA in Solvent Extract	Treatment	Mortality (%)	Degree of Toxicity
*A. westerdijikiae*	1.19 ng/mL	Untreated	55%	T
		Cold plasma treatment	21.66%	ST
*A. steynii*	1.23 ng/mL	Untreated	76.66%	T
		Cold plasma treatment	33.33%	ST
*A. niger*	1.57 ng/mL	Untreated	88.33%	T
		Cold plasma treatment	10%	ST
*A. versicolor*	1.27 ng/mL	Untreated	50%	T
		Cold plasma treatment	16%	ST

OTA toxicity was rated either as nontoxic (NT), slightly toxic (ST), toxic (T), very toxic (VT).

## Supplementary Materials

The following are available online at https://www.mdpi.com/2072-6651/11/6/337/s1, Table S1: raw data.
